# Efficacy of a Standardized Low-Dose Insulin Infusion Protocol in the Emergency Stabilization of Diabetic Dogs

**DOI:** 10.3390/vetsci12100968

**Published:** 2025-10-10

**Authors:** Franco González, Felipe Díaz, Ismael Pereira, Daniela Siel

**Affiliations:** Escuela de Medicina Veterinaria, Facultad de Medicina y Ciencias de la Salud, Universidad Mayor, Santiago 8580000, Chile; felipe.diaz@umayor.cl (F.D.); ismael.pereira@umayor.cl (I.P.); daniela.siel@umayor.cl (D.S.)

**Keywords:** glycemia, canine, ketoacidosis, hyperglycemic syndrome

## Abstract

Background: Continuous insulin infusion protocols are essential for managing decompensated diabetic dogs, but comparative data between variable and fixed infusion rates are limited. Methods: This prospective observational study evaluated the glycemic response of 21 diabetic dogs treated with a fixed-dose continuous-rate infusion (CRI) of regular insulin at 0.05 IU/kg/h for 12 h. Capillary blood glucose was measured hourly. Statistical analyses included Wilcoxon signed-rank tests, Friedman test, Mann–Whitney U, and Kruskal–Wallis tests. Results: A significant reduction in glucose concentration occurred during the first five hours of infusion (*p* < 0.0001), followed by a stabilization phase with no further significant changes. No differences in glycemic response were found by sex or breed. The protocol was well tolerated, with no hypoglycemic events observed. Conclusions: A fixed-dose CRI of 0.05 IU/kg/h offers a safe and effective option for acute glycemic control in diabetic dogs, including those with early ketoacidosis. The standardized approach simplifies management without compromising efficacy and supports its inclusion in emergency treatment protocols.

## Simple Summary

Diabetes mellitus in dogs can progress to an acute decompensated state, characterized by severe hyperglycemia, dehydration, ketosis, and, in some cases, ketoacidosis hyperosmolar hyperglycemic syndrome (HHS). Continuous intravenous insulin infusion is commonly used in emergency management, yet dosing protocols vary between protocols. This study evaluated the effects of a fixed-dose insulin infusion protocol (0.05 IU/kg/h) over 12 h in decompensated diabetic dogs. Capillary blood glucose was monitored hourly, and the influence of sex and breed on glycemic response was analyzed. We observed a marked glucose reduction within the first five hours, followed by stabilization. The protocol was safe, with no hypoglycemic episodes, and sex or breed did not significantly affect glucose response. These findings support the use of a standardized, fixed-rate infusion protocol as a practical and effective option for managing diabetic emergencies in veterinary practice.

## 1. Introduction

Diabetes mellitus (DM) is a prevalent endocrine disorder in dogs, characterized by persistent fasting hyperglycemia resulting from a deficiency in insulin production or action, often due to pancreatic beta-cell destruction or dysfunction [1]. In its most severe form, DM may progress to a decompensated state marked by clinical signs such as dehydration, vomiting, lethargy, and anorexia, and can culminate in diabetic ketoacidosis (DKA) and hyperosmolar hyperglycemic syndrome (HHS) [2]. DKA is a life-threatening metabolic emergency caused by uncontrolled ketogenesis, metabolic acidosis, and electrolyte disturbances that require prompt recognition and aggressive management [3].

The management of decompensated diabetic patients and DKA is multifaceted and includes intravenous fluid resuscitation, correction of electrolyte imbalances, and, critically, the administration of insulin to suppress ketogenesis and reduce hyperglycemia [4]. Among the different approaches to insulin therapy, the continuous-rate infusion (CRI) of regular insulin has gained widespread use due to its ability to provide stable plasma insulin concentrations and facilitate a gradual reduction in blood glucose levels without triggering hypoglycemia [5]. The CRI protocol is often preferred over intermittent dosing because of its more predictable pharmacokinetics and physiologic modulation of glycemia [6].

Two main strategies exist for insulin CRI protocols in veterinary medicine: variable-rate infusion (VRI), where insulin dosage is adjusted frequently based on blood glucose readings, and fixed-rate infusion (FRI), where a constant insulin dose is administered over time, commonly ranging from 0.05 to 0.1 IU/kg/h [7,8]. While VRI offers individualized control, it requires intensive monitoring and mathematical recalculations that may increase the risk of dosing errors in busy clinical settings [9]. On the other hand, FRI protocols simplify the management process, allow easier standardization, and may reduce clinician workload while achieving comparable clinical outcomes [10].

Multiple studies have evaluated the clinical efficacy and safety of these protocols. Macintire (1993) first reported the effectiveness of low-dose CRI of regular insulin in managing canine DKA, demonstrating that this method provided a gradual decline in blood glucose without hypoglycemic events [11]. More recently, Gant et al. (2023) [10] compared FRI and VRI protocols in a prospective, randomized study involving 26 dogs and cats with DKA. They reported similar times to resolution of ketonemia between groups, but animals treated with FRI had shorter hospitalization periods, suggesting potential benefits in efficiency and cost-effectiveness [12].

Despite its practical advantages, the use of FRI protocols in diabetic dogs without DKA but in a decompensated state, such as those presenting with severe hyperglycemia, vomiting, or dehydration, remains less studied [13]. In these patients, where the metabolic crisis is not yet characterized by profound ketosis or acidosis, an early intervention with FRI may prevent progression to full DKA and stabilize glycemic levels without requiring aggressive adjustments [14]. Moreover, standardizing the dose at 0.05 IU/kg/h based on extrapolated data from both experimental models and clinical observations has shown a favorable balance between glycemic control and safety [15].

However, variability in the duration of treatment, timing of glucose nadir, and inter-individual response to insulin remains a challenge. For instance, while some dogs achieve stable glycemia within the first few hours of infusion, others may exhibit delayed or exaggerated responses, underscoring the need for frequent glucose monitoring and further refinement of the protocol [16]. Additionally, most existing studies focus primarily on DKA, limiting the generalizability of findings to non-ketotic hyperglycemic dogs [17].

Therefore, further investigation is needed to define the glycemic kinetics, efficacy, and safety of FRI insulin protocols specifically in dogs with non-ketotic, decompensated diabetes. In this context, the present study aims to evaluate the glycemic response to a fixed-dose insulin CRI protocol at 0.05 IU/kg/h in diabetic dogs presenting in a decompensated state with or without DKA. Capillary blood glucose was monitored hourly for 12 h to characterize the temporal pattern of glycemic decline and assess the protocol’s clinical utility. These findings may contribute to the optimization of insulin infusion protocols in emergency management of diabetic dogs and support the development of standardized treatment guidelines.

## 2. Material and Methods

This was a prospective observational study conducted at Medivet Veterinary Hospital, Santiago, Chile, between 2019 and 2024. The objective was to evaluate the glycemic response to a continuous intravenous insulin infusion protocol in diabetic dogs.

### 2.1. Inclusion Criteria

Dogs eligible for inclusion were those diagnosed with diabetes mellitus and admitted in a decompensated diabetic state, characterized by clinical signs such as dehydration and/or vomiting. Evaluation for diabetic ketoacidosis (DKA) or hyperosmolar hyperglycemic syndrome (HHS) was performed through detection of urinary ketone bodies and/or measurement of serum β-hydroxybutyrate. Patients of any breed, sex, or age were considered. DKA was defined as the presence of ketonemia > 2.0 mmol/L and/or strong ketonuria combined with clinical signs of acid-base disturbance; those considered non-ketotic were dogs with hyperglycemia without detectable ketonemia or ketonuria [11,13]. Patients with concurrent renal, hepatic, or pancreatic disease were excluded. Pancreatitis was ruled out based on clinical signs and normal canine-specific lipase values (Spec cPL < 400 μg/L; IDEXX^®^) [14].

The study population included 21 dogs: 12 females (spayed), and 9 males (neutered). The represented breeds were 13 mixed-breed dogs, 3 Poodles, 2 Beagles, 1 Golden Retriever, 1 Samoyed, and 1 Schnauzer.

### 2.2. Insulin Protocol and Monitoring

All patients received continuous intravenous infusion of regular insulin at a fixed rate of 0.05 IU/kg/h. Capillary blood glucose was measured hourly for 12 h using a validated handheld glucometer (Accu-Chek Instant^®^, Roche, Basel, Switzerland) [15]. No dog received subcutaneous insulin on the day of presentation; of the 21 dogs included, 9 were newly diagnosed diabetics, while 12 had a prior diagnosis and had received insulin therapy before admission. Among the previously treated patients, none had received long-acting insulin formulations such as glargine or detemir on the day of hospitalization. The most commonly used insulin prior to admission was NPH (Insulatard^®^). Rehydration was initiated prior to insulin administration using Ringer Lactate, calculated based on an estimated dehydration of 5% of body weight plus maintenance requirements, administered over the first 6 h. Potassium chloride supplementation was provided as needed. No glucose supplementation was administered during the first 12 h. Supportive medications, including maropitant, antibiotics, or others, were used at the clinician’s discretion. Dogs were offered food once they were clinically stable and able to eat voluntarily. A gastrointestinal prescription diet (Hill’s Prescription Diet i/d^®^) or a homemade bland diet was provided.

### 2.3. Statistical Analysis

All statistical analyses were performed using Stata Statistical Software: Release 13 (StataCorp, 2013). The normality of glucose distribution at each hourly time point was assessed using the Shapiro–Wilk test. As the assumption of normality was not met in most time points (*p* < 0.05), non-parametric methods were selected.

To assess potential differences in glycemic response based on sex and breed, non-parametric tests were applied. The Mann–Whitney U test was used to compare mean blood glucose concentrations between sexes, while the Kruskal–Wallis test was used to evaluate differences among breed groups. A *p*-value < 0.05 was considered statistically significant.

Pairwise comparisons between each consecutive hour were conducted using the Wilcoxon signed-rank test to detect significant changes in blood glucose concentrations over time. Additionally, a Friedman test—used as a non-parametric equivalent to repeated-measure ANOVA—was applied to evaluate the overall effect of time on glucose concentrations throughout the 12 h infusion period. A *p*-value < 0.05 was considered statistically significant for all analyses.

### 2.4. Population Characteristics

A total of 21 diabetic dogs fulfilled the inclusion criteria and were enrolled in the study. Patients represented various breeds, sexes, and ages, and all were admitted due to a decompensated diabetic state. The median age was 9.2 years (range: 4–15 years), and the median body condition score (BCS) was 5/9 (range: 3–7/9). Eight dogs presented with vomiting, and five dogs were hyperglycemic and moderately dehydrated (estimated at 5%) without additional systemic signs such as vomiting, lethargy, or anorexia. Seven dogs were diagnosed with diabetic ketoacidosis based on urinary ketones and/or elevated serum β-hydroxybutyrate levels, eleven dogs were non-ketotic on admission, and three lacked complete ketone data. Biochemical, hematological, and urinary analyses considered concurrent organ dysfunction unlikely based on clinical and laboratory findings.

## 3. Results

**Figure 1:**
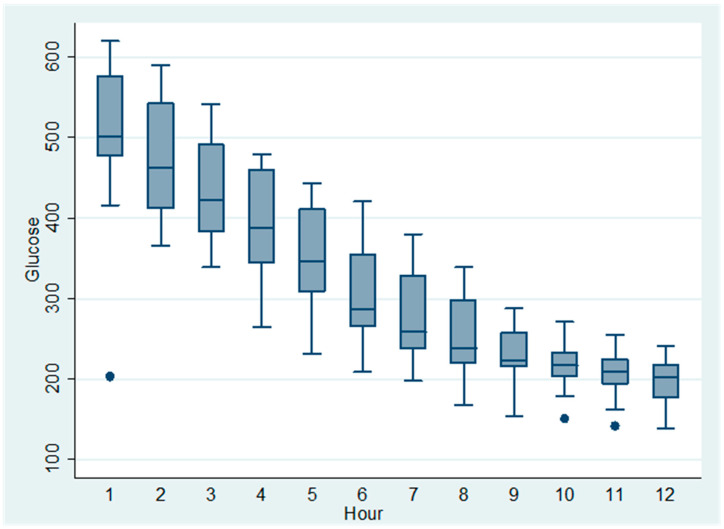
Temporal progression of capillary blood glucose levels in 21 diabetic dogs receiving continuous intravenous infusion of regular insulin at a fixed rate of 0.05 IU/kg/h. Each boxplot represents median, interquartile range (IQR), and range (whiskers) of glycemic values at each hourly time point over 12 h. Outliers were defined as values exceeding 1.5 times the interquartile range (IQR). Each dot represents an individual glucose measurement from a single dog at the corresponding time point.

All dogs received a continuous-rate infusion (CRI) of regular insulin at 0.05 UI/kg/h, and capillary blood glucose was monitored hourly for 12 h. The initial mean glucose concentration at Hour 1 was 590 mg/dL (±SD), decreasing progressively to 220 mg/dL by Hour 12 (Table 1). The temporal trend of glucose reduction is visually summarized in Figure 1, which illustrates a marked early decrease followed by glycemic stabilization.

Beta-hydroxybutyrate levels and venous pH were evaluated at admission and again 6 h after insulin infusion. Both parameters showed a decreasing trend, with median β-hydroxybutyrate decreasing from 2.8 mmol/L (range: 1.5–4.6) to 1.2 mmol/L (range: 0.4–2.3), and pH increasing from 7.25 (range: 7.10–7.35) to 7.32 (range: 7.25–7.40) (Table S1).

Pairwise comparisons using the Wilcoxon signed-rank test demonstrated statistically significant reductions in blood glucose during the first five hours: Hour 1 vs. Hour 2 (W = 0.0, *p* = 0.0001), Hour 2 vs. Hour 3 (W = 0.0, *p* < 0.00001), Hour 3 vs. Hour 4 (W = 0.0, *p* < 0.00001), and Hour 4 vs. Hour 5 (W = 0.0, *p* < 0.00001). From Hour 6 onward, no further statistically significant changes were observed (*p* > 0.05), suggesting that the treatment effect reached a plateau.

To evaluate the overall effect of the 0.05 UI/kg/h insulin infusion on blood glucose across the full 12 h period, a Friedman test was performed using only dogs with complete glycemic profiles (*n* = 15). The result indicated no statistically significant global change over time (χ^2^ = 11.0, df = 11, *p* = 0.443), likely reflecting the strong initial reduction followed by glycemic stabilization, which may obscure overall significance in non-parametric repeated-measure analysis.

No statistically significant differences were found in glycemic response based on sex or breed. The Mann–Whitney U test comparing mean blood glucose concentrations between females (*n* = 12) and males (*n* = 9) yielded a U value of 65.0 (*p* = 0.455). Similarly, the Kruskal–Wallis test comparing mean glycemia among different breed groups revealed no significant differences (H = 4.41, *p* = 0.492). These findings suggest that, within this population, sex and breed did not significantly influence the glycemic response to the insulin infusion protocol (Table 2).

These findings support the notion that continuous insulin infusion at this dosage induces an early, clinically significant glucose-lowering effect in decompensated diabetic dogs, particularly within the first five hours of treatment.

## 4. Discussion

The present study evaluated the glycemic response of decompensated diabetic dogs to a fixed-dose continuous-rate infusion (CRI) of regular insulin at 0.05 IU/kg/h over a 12 h period. The main findings indicate that this protocol achieved a rapid and significant reduction in capillary blood glucose concentrations during the first five hours of treatment, followed by a stabilization phase from Hour 6 onward. These results provide relevant clinical insights into the application of fixed-rate insulin infusions in emergency management of diabetic dogs, particularly those presenting with severe hyperglycemia, dehydration, or early-stage ketoacidosis. For the purposes of this study, early DKA was defined as mild to moderate ketosis with preserved hydration and without marked acidosis (venous pH > 7.25) in dogs presented within 24 h of onset of clinical signs.

Our data support prior observations that fixed-rate CRI protocols can effectively lower glycemia in diabetic patients without inducing hypoglycemia, especially when monitored intensively [6,9,13]. Previous investigations, such as those by Macintire [6] and Gant et al. [18], have demonstrated the efficacy of low-dose insulin CRIs in dogs with diabetic ketoacidosis (DKA), achieving controlled glycemic decline and resolution of ketonemia. However, fewer studies have focused on dogs presenting with non-ketotic decompensation, despite the clinical relevance of this subgroup. Our study addresses this gap and shows that early glycemic control can be achieved safely using a fixed-dose CRI protocol, potentially preventing progression to full DKA. For the purposes of this study, early glycemic control was defined as a reduction in capillary blood glucose of more than 50% from baseline within the first 6 h of insulin infusion.

The fixed dose of 0.05 IU/kg/h was chosen based on previous veterinary recommendations [13,14,15] and was shown to be adequate for producing a gradual decrease in blood glucose without excessive variability. The initial glycemic drop observed within the first hours corroborates findings from Palus et al. [13] and Wildermuth et al. [15], who reported that this dosage minimizes the risk of insulin-induced hypoglycemia while ensuring effective glucose suppression. The observed stabilization of glucose values from Hour 6 onward likely reflects a homeostatic plateau where endogenous insulin sensitivity and fluid resuscitation contribute to metabolic equilibrium.

A novel aspect of our study was the statistical comparison of glycemic response by sex and breed. Although Table 2 shows small numeric differences in mean blood glucose concentrations across groups, statistical analysis did not reveal significant differences. These findings suggest that, at least in the short-term administration of insulin via CRI, neither sex nor breed exerts a clinically relevant influence on glucose-lowering response. This observation is consistent with reports by Rand et al. [12] and Behrend [14], who emphasized that inter-individual response is more likely governed by factors such as hydration status, counter-regulatory hormones, and severity of hyperglycemia, rather than inherent biological sex or breed.

It is noteworthy that although seven dogs in our cohort were diagnosed with DKA, the standardized approach allowed for inclusion and simultaneous monitoring of dogs with and without ketosis. This integrated analysis reflects real-world scenarios where diabetic crises often exist on a spectrum of severity [7,14,15]. Moreover, hourly monitoring over 12 h provided a robust dataset for evaluating the temporal kinetics of glycemic decline, enabling clinicians to anticipate inflection points for stabilization and potential adjustment.

One limitation of the study is the relatively small sample size and lack of a control group receiving variable-rate infusion (VRI), which could allow for direct protocol comparisons. Nevertheless, the prospective and standardized design, combined with intensive glucose monitoring, strengthens the internal validity of our findings. Future multicenter studies with larger cohorts should aim to compare fixed and variable protocols, assess long-term outcomes, and explore the impact of adjunctive treatments (e.g., electrolyte correction, nutritional support) on glycemic dynamics.

In conclusion, this study confirms that a fixed-dose CRI of regular insulin at 0.05 IU/kg/h is a clinically useful and safe strategy for acute glycemic management in decompensated diabetic dogs. The early glycemic decline and subsequent stabilization observed support the utility of this protocol in veterinary emergency settings. Our findings encourage the adoption of fixed-dose protocols with adequate monitoring as part of standardized guidelines for managing diabetic emergencies in dogs.

## Figures and Tables

**Table 1:** Glycemic parameters during insulin infusion over 12 h. Values are presented as median and range. Wilcoxon signed-rank test used for pairwise comparisons.

Hour	Median (mg/dL)	Range (mg/dL)	*p* vs. Previous Hour
1	506	416–620	–
2	460	366–590	0.0001
3	416	354–510	<0.00001
4	378	323–470	<0.00001
5	345	274–442	<0.00001
6	310	211–399	NS
7	296	244–355	NS
8	266	220–317	NS
9	232	188–288	NS
10	210	166–246	NS
11	198	154–232	NS
12	190	142–220	NS

Summary of glycemic parameters recorded hourly during the 12 h continuous-rate insulin infusion (0.05 IU/kg/h) in diabetic dogs. Values represent median and range (minimum–maximum) of capillary blood glucose concentration (mg/dL). Wilcoxon signed-rank test was used for comparisons between consecutive hours.

**Table 2:** Mean blood glucose by sex and breed. Data are shown as mean ± SD for exploratory purposes. Non-parametric distribution.

Group	*n*	Mean (mg/dL)	SD	Range (mg/dL)
Female	12	344	78	202–474
Male	9	329	76	215–457
Mixed Breed	13	332	79	202–474
Poodle	3	366	57	301–417
Beagle	2	370	16	359–382
Golden Retriever	1	287	–	–
Samoyed	1	394	–	–
Schnauzer	1	270	–	–

Summary of mean capillary blood glucose concentrations over 12 h of continuous insulin infusion, grouped by sex and breed. SD = standard deviation.

## Data Availability

The original contributions presented in this study are included in the article/Supplementary Material. Further inquiries can be directed to the corresponding author.

## Supplementary Materials

The following supporting information can be downloaded at https://www.mdpi.com/article/10.3390/vetsci12100968/s1: Table S1: Changes in Beta-Hydroxybutyrate and Venous pH Values at Baseline and 6 Hours After Insulin Infusion.
