# Supplementary material for: Efficacy of a Standardized Low-Dose Insulin Infusion Protocol in the Emergency Stabilization of Diabetic Dogs
Source: Vet Sci. 2025 Oct 10;12(10):968. doi: 10.3390/vetsci12100968 (PMC12567954; doi:10.3390/vetsci12100968)
Supplement: Supplementary file 1 [file vetsci-12-00968-s001.zip › vetsci-3714582-supplementary.pdf]

Supplementary Table S1. Changes in Beta-Hydroxybutyrate and Venous pH Values at Baseline and 6 Hours After Insulin Infusion

**Parameter**	**Timepoint**	**Median**	**Range**	**Unit**
Beta-hydroxybutyrate	Baseline	2.8	1.5–4.6	mmol/L
Beta-hydroxybutyrate	6 h	1.2	0.4–2.3	mmol/L
Venous pH	Baseline	7.25	7.10–7.35	
Venous pH	6 h	7.32	7.25–7.40	
